# Supplementary material for: Rapid turnover of life-cycle-related genes in the brown algae
Source: Genome Biol. 2019 Feb 14;20:35. doi: 10.1186/s13059-019-1630-6 (PMC6374913; doi:10.1186/s13059-019-1630-6)
Supplement: Supplementary file 3 — Protein alignments, corresponding Gblocks html file and CDS sequences for the lineage conserved gametophyte-biased genes. (ZIP 2109 kb) [file 13059_2019_1630_MOESM3_ESM.zip › Con_GA_alignments/Ec-06_008130.aa_ali.fasta-gb.htm]

Ec-06\_008130.aa.fasta.trans.aa\_ali.fasta


## Gblocks 0.91b Results

Processed file: **Ec-06\_008130.aa.fasta.trans.aa\_ali.fasta**  
Number of sequences: **2**  
Alignment assumed to be: **Protein**  
New number of positions: **175** (selected positions are underlined in blue)

```
                         10        20        30        40        50        60
                 =========+=========+=========+=========+=========+=========+
Ec-06_008130     ------------------------------------------------------------
MSTRG.48023.1    MYPRLVTLTIEQIWNGINGVVRVVFVQATTSGLSGEVRGFSLRAHVTQYIGDVQPAVVLC
                                                                             


                         70        80        90       100       110       120
                 =========+=========+=========+=========+=========+=========+
Ec-06_008130     -------------------------------------MVVFGRANRDDLSKLFLGKDAVA
MSTRG.48023.1    TFHILQHSMRFRTTDRCIRAISTMSLFQARDLCERPHGLIFGRAKRDDLAKLFLGKDAVA
                                                         ####################


                        130       140       150       160       170       180
                 =========+=========+=========+=========+=========+=========+
Ec-06_008130     YVKRNTPGPGTYELRWHRPVFQFPTREPGITIGGPLANVDRVQTGPLGAAYRSDTPGPG-
MSTRG.48023.1    YVTRTSPGPGTYELGYQRPVFKFLSHEPGPLIGGPLANVDRVQTGPLGEAYRSDTPGPGA
                 ########################################################### 


                        190       200       210       220       230       240
                 =========+=========+=========+=========+=========+=========+
Ec-06_008130     -----------------------FGSEWVLLSSGVGIG----------IGAWASE-----
MSTRG.48023.1    YHETDVRTASTRRRPGRDEFFSRAGSTSTSSWSGTEFGRASTEGCKRPITGRGGEGDEGG
                                         ##############                      


                        250       260       270       280       290       300
                 =========+=========+=========+=========+=========+=========+
Ec-06_008130     ---YQGIR--------------------------------------------------TG
MSTRG.48023.1    SQFYPGVRSCHGEEVDGPFSLSPFRFVDNQGHDSWQLPLLGPRRPASTPSRRKTRASQSG
                                                                            #


                        310       320       330       340       350       360
                 =========+=========+=========+=========+=========+=========+
Ec-06_008130     QLAAALTGLDTPPINTIQKQSSGFGNVSVSWHLSSAARPVTTFGTGTREQAGQALAPGFK
MSTRG.48023.1    RLAKGPKAKDQGKIGSASSLSAGYS--QASWQSSLASRPVTTFGMGSREQAGQVLAPGFK
                 #######################      ###############################


                        370       380       390       400       410
                 =========+=========+=========+=========+=========+=======
Ec-06_008130     GYASRCNIANPGPGAYRNVPGSMGNQVLEIEDSFVAVDECRPLASTHDFLFISGERI
MSTRG.48023.1    GCAPRSTIGNPGPGAYRHVPGSLGPQVQGNT--------------------------
                 ###########################
```

```
Parameters used
Minimum Number Of Sequences For A Conserved Position: 2
Minimum Number Of Sequences For A Flanking Position: 2
Maximum Number Of Contiguous Nonconserved Positions: 8
Minimum Length Of A Block: 10
Allowed Gap Positions: None
Use Similarity Matrices: Yes
```

```
Flank positions of the 4 selected block(s)
Flanks: [101  179]  [205  218]  [300  323]  [330  387]  

New number of positions in Ec-06_008130.aa.fasta.trans.aa_ali.fasta-gb:  175  (41% of the original 417 positions)
```
